# Emotional Awareness Correlated With Number of Awakenings From Polysomnography in Patients With Myalgic Encephalomyelitis/Chronic Fatigue Syndrome—A Pilot Study

**DOI:** 10.3389/fpsyt.2020.00222

**Published:** 2020-03-26

**Authors:** Indre Bileviciute-Ljungar, Danielle Friberg

**Affiliations:** ^1^Department of Clinical Sciences, Karolinska Institutet, Danderyd University Hospital, Stockholm, Sweden; ^2^Department of Surgical Sciences, Uppsala University, Uppsala, Sweden

**Keywords:** myalgic encephalomyelitis/chronic fatigue syndrome, sleep, awakenings, alexithymia, emotional awareness

## Abstract

**Introduction:**

Unrefreshing sleep is one of the diagnostic criteria in myalgic encephalomyelitis/chronic fatigue syndrome (ME/CFS), which could be explained by sleep disorders, for example obstructive sleep apnea, reported in our previous study with polysomnography. Our previous findings also indicate difficulties in emotional regulation when measuring alexithymia by TAS-20 (Toronto Alexithymia Scale) and level of emotional awareness by LEAS (Level of Emotional Awareness Scale) in ME/CFS patients. However, the reasons for this are unknown. The purpose of this study was to investigate correlations between data from subjective emotional regulation and polysomnography.

**Methods:**

Twenty-three ME/CFS patients (5 men and 18 women) of mean age 43, and 30 matched healthy controls (9 males and 21 women) of mean age 45, filled in TAS-20, LEAS, and Hospital Depression and Anxiety Scale (HADS). A polysomnography was performed on patients but not on healthy controls. Thus, values of normal population were used for sleep evaluation in ME/CFS patients.

**Result:**

There were significant differences between patients and controls in several aspects of emotional regulation, for example LEAS-self and LEAS-total. Seventy percent of the patients had increased numbers of awakenings (shifts from any sleep stage to awake), 22% had obstructive sleep apneas, and 27% had periodic limb movements. Correlation analysis showed that number of awakenings significantly correlated with LEAS-self and LEAS-total, p < 0.01, respectively. There were no other significant correlations.

**Conclusion:**

This pilot study demonstrated significant correlations between reduced emotional awareness and number of awakenings in polysomnography. Future studies with larger cohorts need to be conducted.

## Introduction

Myalgic encephalomyelitis/chronic fatigue syndrome (ME/CFS) is characterized by pathological fatigue and fatigability and a number of symptoms, presented over a 6 months period. Patients also report pain, gastrointestinal symptoms, and cognitive difficulties (1). They also complain about the feeling of malaise and fever, both worsening with effort, as well as sore throat and/or lymph nodes (1). The prevalence varies from 1.2%, when the stricter Canadian criteria are applied, to 2.6%, when the broad Oxford criteria are used (2, 3). Sleep disturbances is another important criterion of ME/CFS. However, the exact definition of sleep disturbances in case definition guidelines for ME/CFS is not clear (4). Subjectively reported “non-restorative sleep” or “waking non-rested in the morning” is good enough to classify the presence of sleep disturbances. Our recent study of a total of 381 patients referred for ME/CFS showed that 20.5% (n = 78) of patients fulfilled the criteria for further investigation with full-night polysomnography (5). Thus, patients had increased excessive daytime sleepiness and/or tiredness. Among them, 31 (40.3%) patients were diagnosed with obstructive sleep apnea (OSA), 7 (8.9%) patients with periodic limb movement disorder, and 32 (41.0%) patients with restless legs syndrome. Taken together, among those reporting pathological sleep symptoms, 69.3% of patients had one or more sleep disorders. Further, a majority (84%) had an increased “number of arousals” per sleep hour (mean 8.7, SD 5.1). However, these arousals were actually a shift from any sleep stage to waking, and are in the present study called “number of awakenings”. A majority of these awakenings were probably caused by the sleep disorders. Other authors have speculated that sleep disorders in general contribute not only to fatigue (6) and cognition failure (7), the key symptoms in ME/CFS, but also to emotional regulation (8). Since emotional regulation starts with identification of emotions and situations related to these emotions (8), the emotional awareness seems to be crucial in the process of emotional regulation. Our recent study indicated that ME/CFS patients had increased alexithymia by scoring in Toronto Alexithymia Scale (TAS-20) and lower levels of emotional awareness by scoring in Level of Emotional Awareness Scale (LEAS), as compared to healthy controls (9).

Alexithymia can be described as a reduced capacity to conceptualize emotional information, related to both verbal and non-verbal stimuli (10). This leads to disturbances of affect regulation (11). For example, alexithymic individuals have difficulties identifying and describing their own or others' feelings (10) and have an externally-oriented thinking style with a scarcity of fantasy life (12, 13). Alexithymia might be considered as an original personality trait (14) or may develop as a result of a stressful situation as well as medical/psychiatric illness (15). Emotional awareness is another construct related to alexithymia but does not include externally-oriented thinking (16). Both measures show a significant but weak negative correlation (17).

Sleep disorders such as obstructive sleep apnoea (OSA) and periodic limb movement disease are well-known causes of sleep fragmentation, with awakenings and arousals, noted during polysomnography (18).

The aim of this study was to investigate correlations between the alexithymia (TAS-20) and the level of emotional awareness (LEAS), as well as depression and anxiety [Hospital Anxiety and Depression Scale (HADS)], with objective sleep parameters from polysomnography in patients with ME/CFS.

## Methods

### Participants

Twenty of the 23 ME/CFS patients were included from our recently published cohorts (5, 9). Three additional patients who underwent polysomnography, but whose results from questionnaires had not previously been analyzed, were also included. The inclusion criteria for the present study was diagnosis of ME/CFS given by a multidisciplinary team, completed TAS-20 and LEAS, and a full-night polysomnography. Our criteria for performing polysomnography (PSG) were excessive daytime sleepiness, evaluated by Epworth Sleepiness Scale (ESS) ≥ 10, and/or often or always tired during mornings and/or daytime. A full-night polysomnography was performed at the Department of Otorhinolaryngology (ORL), Karolinska University Hospital. Afterwards, the results of the manual polysomnography-scorings were analyzed by a specialist in sleep medicine (Danielle Friberg).

All 23 patients had entered a clinical ME/CFS project at Danderyd University Hospital, Stockholm during the period from 2011 to 2013. During 2011–2016, the unit at the Danderyd University Hospital served as a public healthcare tertiary clinic for patients with chronic fatigue. The majority of patients were referred from primary healthcare clinics, psychiatry, and neurology departments within Stockholm County. The majority of patients were already thoroughly investigated and had a long history of fatigue. Their fatigue was not explained by other primary diseases and they were refereed to ME/CFS clinic for further investigation.

The diagnosis of ME/CFS was made in accordance with the Centers for Disease Control (19) and/or Canadian criteria (1), summarized in **Table 1**. During 2011–2012, patients were evaluated by a team: a clinician, who was a specialist in rehabilitation medicine or neurology; a psychologist, who performed a standardized psychiatric interview and neuropsychological testing; a physiotherapist, an ergotherapist, a social worker, and a nurse. The laboratory tests were performed for all patients in order to exclude other pathological conditions, since ME/CFS is considered to be an exclusionary diagnosis. The majority of patients were also referred to brain magnetic resonance imaging to exclude brain disorders. Due to administrative changes, the complete team-based evaluation was terminated 2013. Thus, the physician and psychologist performed clinical evaluation during a 90-minutes visit.

**Table 1:** Comparison of set of criteria for ME/CFS according to CDC/Fukuda (19) and Canada (1), both coded as G93.3 in ICD-10.

Symptoms	Chronic fatigue syndrome (19).	Myalgic encephalomylitis (1) G93.3. Abridged.
Exhaustion	No required symptom but included as an additional symptom.	A) Post-exertional malaise, with hallmark features: 1. Physical and/ or cognitive fatigability; 2. Post-exertional symptom exacerbation; 3. Recovery period is prolonged, usually taking 24 h or longer.
Fatigue	A) Severe chronic fatigue.	B) Physical and mental fatigue.
Additional symptoms	B) At least four of the following symptoms: B1) substantial impairment in short-term memory or concentration; B2) headaches of a new type, pattern, or severity; B3) tender lymph nodes; B4) unrefreshing sleep; B5) post-exertional malaise lasting more than 24 hours; B6) muscle pain; B7) pain in the joints without swelling or redness; B8) a sore throat that is frequent or recurring.	C) Sleep dysfunction. D) Pain. E) Neurological/cognitive manifestations: Two or more (i.e., impairment of concentration and short-term memory consolidation, difficulty with information processing, overload phenomena: cognitive, sensory—e.g., photophobia and hypersensitivity to noise etc.) F) At least one symptom from two of the following categories: F1) Autonomic manifestations (i.e., orthostatic intolerance—NMH, POTS etc.; F2) Neuroendocrine manifestations (i.e., loss of thermostatic instability—subnormal body temperature and marked diurnal fluctuation, sweating episodes, recurrent feelings of feverishness and cold extremities; worsening of symptoms with stress etc.); F3) Immune manifestations (i.e., tender lymph nodes, recurrent sore throat, recurrent flu-like symptoms, etc.)
Functional impairment	Significantly interferes with daily activities and work.	Substantially reduced activity level as compared to premorbid level.
Duration	Symptoms should have existed for at least 6 months.	Symptoms should have existed for at least 6 months.
Exclusion	Medical disorders primarily explaining the fatigue. Several psychiatric disorders.	Medical disorders primarily explaining the symptoms. Primary psychiatric disorders.
Precipitating factor	Infection in up to 75%	Infection in up to 75%

ME/CFS, myalgic encephalomyelitis/chronic fatigue syndrome; NMH, neurally mediated hypotension; POTS, postural orthostatic tachycardia syndrome.

The group of healthy controls (n = 30) had previously participated in studies on emotional awareness (5) and health-related life quality (20). The healthy control group was recruited by using a convenience sample of family, friends, and co-workers. Healthy controls should not have had current or previous diagnosis of ME/CFS and other comorbidities such as psychiatric disorders, chronic pain, allergies/asthma, sleep disorders, hypothyreosis, heart disorders, or be on the sick-leave. They also scored high for health-related quality of life (20).

### Questionnaires

- The ESS—a self-administered questionnaire measures daytime sleepiness, the maximum score is 24 points (21). The patients also responded to a non-validated questionnaire, confirming the clinical symptoms for further indication to PSG.
- The HADS screens the levels of anxiety and depression, the maximum score is 21 point for each measure (22).
- The TAS-20 is a self-reported questionnaire measuring alexithymic traits. Twenty items are rated using a 5-point Likert scale. The items are divided into three subscales: 1) Difficulty Identifying Feelings (DIF); 2) Difficulty Describing Feelings (DDF); and 3) Externally Oriented Thinking (EOT). The TAS-20 has demonstrated good internal consistency with test–retest reliability in the Swedish population (23). In the present study, the Cronbach´s alpha coefficient for TAS-20 subscales was 0.8.

LEAS is an observer-rated measure of emotional awareness (24). The shortened 10-item version of the LEAS (LEAS-A) was applied. The test consists of 10 descriptions of emotion-provoking interactions between two people. Participants were asked to describe how they would feel as a protagonist in each scene and how “the other person” would feel. Answers were quantified according a manual of scoring rules. Scores from 0–5 were assigned for the categories “self,” “other,” and “total,” with lower scores reflecting a lower level of emotional awareness. Maximum “total” scores of 50 and “self” or “other” scores of 40 were possible. A score of 0 was given when the subject either gave no answer or did not describe thoughts (i.e., *I feel that it is expensive*.). A score of 1 was given when bodily sensations were described (i.e., *I would feel fatigued*.). A score of 2 was given to an action tendency or an undifferentiated affect state (i.e., *I would like to run away. My friend would feel good.)*. A score of 3 was given when a single emotion was described (i.e., *I would feel happy*.). A score of 4 was given to blends of emotions (i.e., *I would feel angry and a bit of sadness*.). A score of 5 was given to multiple blends of emotions (i.e., *I would feel disappointed. But if someone else won, I would be happy that it was my friend. My friend will be proud and happy, but also concerned about me*.). The Cronbach´s alpha coefficient for LEAS subscales was 0.94.

### Polysomnography

A full-night polysomnography was performed using the Embla technology (Flaga Medical; Reykjavik, Iceland) in a sleep laboratory. PSG recordings were interpreted manually by registered technicians, later on also checked by a specialist in sleep medicine (Danielle Friberg). Due to the fact that a sleep laboratory was used in a day-care unit, the patients were woken at 6 am. Totally, 16 channels were recorded: electroencephalography (EEG) (sensors C3-A2, O1-A2, O2-A1, C4-A1), electrooculography (EOG) (left and right), electromyography (EMG) chin and tibialis (left and right), oronasal flowmetry, respiratory movements (abdomen and thorax), snoring, electrocardiography (ECG), pulse, and body position. PSG parameters of American Academy of Sleep Medicine 2007 for normal values were used for comparisons (25).

The use of clinical data for scientific analysis was considered by the regional ethical review board in Stockholm (Ref. no. 2014/300-31) and approved by Danderyd University Hospital (DS2014-0447).

### Statistical Analysis

Descriptive statistics were presented by percentage, medium (minimum-maximum), and by mean with standard deviation as well as 95% confidence interval, when appropriated due to the qualitative *vs.* quantitative parameters measured. Nonparametric tests (Mann-Whitney U) were used for group comparisons of ordinal data from questionnaires, and parametric independent two-tailed t-tests were used for age. A p-value <0.05 was considered significant for these tests. A non-parametric Spearman correlation test was used to analyze correlations between emotional questionnaires (TAS-20, LEAS, and HADS) and sleep parameters, respectively. Due to multiple analysis, a stricter criterion for statistical significance was used, a p-value less than 0.01 was considered a significant correlation, and p-value less than 0.05 was considered a weak correlation. Cronbach´s alpha was used for internal consistency of TAS-20 and LEAS.

The statistical package SPSS 25.0 was used for coding and analyzing the data.

## Results

### Emotional Status in ME/CFS Patients and Healthy Controls

Twenty-three patients (5 men and 18 women) with a mean age of 43 (SD 12) years, and a mean body mass index (BMI) of 24 (SD 3) were included in the study. Their mean ESS score was 7.6 points and there were 7 (30%) patients with a pathological ESS ≥ 10.

Healthy controls (9 males and 21 women) were 45 years old (SD 12) and there was no difference between the groups concerning age and gender (BMI of controls not measured). Comparisons between ME/CFS patients and healthy controls showed that ME/CFS patients had significantly higher scores in TAS-DIF and total TAS-20, as well as significantly lower values in LEAS-self and LEAS total (**Table 2**).

**Table 2:** Results from three questionnaires concerning alexithymia (TAS-20), emotional awareness (LEAS) and mood (HADS) from ME/CFS patients and healthy controls.

Group	TAS-20 DIF N=21–30	TAS-20 DDF N=21–30	TAS-20 EOT N=21–30	TAS Total N=21–30	LEAS-Self N=23–30	LEAS-Other N=22–30	LEAS-Total N=23–30	HADS Depression N=23–30	HADS Anxiety N=23–30
ME/CFS	16 *** [9–31]	9 [5–17]	16 [9–29]	39* [27–68]	28** [13–33]	24 [11–32]	29** [11–36]	5 *** [1–15]	5 [0–16]
Controls	10.5 [7–19]	8 [5–14]	13 [8–25]	33.5 [20–44]	32 [13–39]	28.5 [11–38]	36 [22 -45]	1 [0–11]	4 [0–9]

DIF, difficulty in identifying feelings; DDF, difficulty in describing feelings; EOT, externally orientated thinking; HADS, Hospital Anxiety and Depression Scale; LEAS, Level of Emotional Awareness Scale; ME/CFS, myalgic encephalomyelitis/chronic fatigue syndrome; TAS-20, Toronto Alexithymia Scale.

Values are presented as median, with minimum and maximum values. Comparison between the groups was performed by using Mann-Whitney test and significance is indicated as following: *p < 0.05, **p < 0.01 and ***p < 0.000.

The clinical emotional status was also scored using the HADS (**Table 2**). Six (26%) patients scored ≥ 10 in HADS depression and 4 (17%) ≥ 10 in HADS anxiety. Among the controls, only 1 (3%) participant scored 11 in HADS depression and no-one scored ≥ 10 in HADS anxiety. The HADS depression index was also significantly higher in ME/CFS patients as compared to controls (**Table 2**).

HADS depression correlated significantly with TAS-20 (r = 0.47, p = 0.000; r = 0.37, p = 0.008 and r = 0.49, p = 0.000; DIF, DDF, and TAS-total, respectively) and LEAS (r = −0.44, p = 0.001; r = −0.36, p = 0.008, and r = −0.51, p = 0.000; LEAS-self, LEAS-other, and LEAS-total, respectively). HADS anxiety had a weak correlation with DDF (r = 0.3, p = 0.02) but with no other TAS/LEAS parameters.

### Objective Sleep Measures in ME/CFS Patients From Polysomnography

**Table 3** summarizes sleep measures from the polysomnography. The majority of patients had reduced total sleep (100%) and increased sleep latency (78%). For more than half of the patients, the percentage of N1 stage was higher (61%) and REM was lower (57%). The number of awakenings was higher in 16 patients (70%), though the total arousal index was impaired only in 5 (22%) of patients. Their mean apnoea-hypopnoea index (AHI) was 2.9 (SD 3.2), and 22% had an AHI > 5, which is the definition of OSA, and 27% had a periodic limb movements index (PLMI) above 5, but only one had a PLM arousal index above 5.

**Table 3:** The results from polysomnography of ME/CFS patients and compared to normal values according to the AASM 2007 (25).

Polysomnography measures	Polysomnography values	Abnormal values according AASM	Number of patients with abnormal values
Total sleep time (min) N=23	291.7 (75.6) [259.0–324.4]	<7 h (420 min)	23 (100%)
		>9 h (540 min)	0
Sleep efficiency (SE) (%) N=23	75.9 (16.2) [68.9–82.9]	85%—decreased	14 (61%)
Sleep latency (min) N=23	36.0 (26.2) [24.7–47.3]	<5 min	1 (4.3%)
		>20 min	18 (78%)
N3 sleep latency (min) N=16	85.9 (53.7) [57.3–114.6]	NA	NA
REM stage latency (min)† N=20	141.0 (73.3) [106.6–175.3]	≤8 min	0
Number of awakenings N=23	4.9 (7.3) [1.7–8.0]		16 (70%) had more than 5
Respiratory Arousal Index N=23	2.3 (3.1) [1.0–3.7]	>5	4 (17%)
Spontaneous Arousal Index N=22	2.0 (1.9) [1.1–2.8]	>15	0
Total Arousal Index N=23	10.5 (10.2) [6.1–14.9]	>15	5 (22%)
N1 duration (%) N=23	15.0 (8.7) [11.2–18.7]	<5%	1 (4.3%)
		>10%	14 (61%)
N2 duration (%) N=23	51.3 (17.6) [43.7–58.9]	<45%	9 (39%)
		>55%	7 (30%)
N3 duration (%) N=23	18.2 (9.1) [14.2–22.2]	<5%	1 (4.3%)
		>25%	5 (22%)
REM duration (%) N=23	12.4 (8.0) [9.0–15.9]	<15%	13 (57%)
		>25%	1 (4.3%)
AHI N=23	2.9 (3.2) [1.5–4.3]	≥5	5 (22%)
Supine position-related AHI N=20	4.2 (9.1) [-0.1–8.5]	≥5	5 (22%)
Central AHI N=23	7.2 (9.4) [3.1–11.2]	≥5	9 (39%)
RERA index N=23	4.9 (7.3) [1.7–8.0]	≥5	6 (26%)
ODI N=22	2.3 (3.7) [0.7–3.9]	≥5	1 (4.3%)
PLMI N=22	5.8 (11.8) [0.6–11.0]	>5	6 (27%)
PLMAI N=22	1.3 (3.9) [−0.5–3.0]	≥5	1 (4.5%)

AASM, American Academy of Sleep Medicine; AHI, apnoea-hypopnoea index; AI, arousal index; ME/CFS, myalgic encephalomyelitis/chronic fatigue syndrome; N1, N2, N3, respectively sleep stage 1, 2, 3; NA, not applicable; ODI, oxygen desaturation index; PLMAI, periodic limb movements arousal index; PLMI, periodic limb movements index; REM, rapid eye movements; RERA, respiratory effort related arousals.

†The cut-off of 8 min for REM stage latency is chosen according to the diagnostic criteria for narcolepsy.

Values are presented as mean with standard deviation in the parenthesis (first row) and 95% confidence interval (second row). The numbers of patients with abnormal values are presented as a total number and a percentage.

### Correlation Analysis

**Table 4** summarizes correlation analysis between polysomnography and emotional parameters TAS-20, LEAS, and HADS. A negative significant correlation was found between LEAS-self and LEAS total with number of awakenings registered by polysomnography (r = −0.6, p = 0.005–0.001). Weak correlations (p < 0.05) were found between DIF TAS-20 and total TAS-20 with percentage of deep sleep (r = −05, p = −0.02) as well as between LEAS-self and LEAS-total with percentage of N1 (r = −0.5, p = 0.02–0.03), respectively.

**Table 4:** Results from Spearman correlation analysis with r-values between sleep parameters from polysomnography and questionnaires TAS-20, LEAS and HADS, respectively, in ME/CFS patients.

	TAS-20 DIF	TAS-20 DDF	TAS-20 EOT	TAS Total	LEAS-Self	LEAS-Other	LEAS-Total	HADS Depression	HADS Anxiety
Total sleep time	0.03	−0.13	−0.22	−0.16	0.12	0.28	0.16	−0.38	−0.36
Sleep efficiency	−0.254	−0.263	−0.318	−0.390	0.07	0.25	0.15	−0.44	−0.43
Sleep latency	−0.18	−0.09	0.17	0.01	0.10	−0.23	0.001	0.30	0.37
REM latency	−0.07	0.01	0.14	0.01	−0.045	−0.19	−0.04	0.32	0.37
N3 latency	−0.05	−0.35	0.04	−0.12	−0.09	−0.43	−0.10	0.47	0.25
N1 %	0.06	0.10	0.39	0.21	−0.49	−0.33	−0.4	0.35	0.16
N2 %	0.44	0.18	0.27	0.39	−0.05	0.17	0.00	−0.26	−0.11
N3 %	−0.44	−0.33	−0.43	−0.51	0.18	0.09	0.26	−0.03	−0.17
REM %	−0.076	−0.140	−0.216	−0.192	0.37	0.24	0.31	−0.27	−0.23
Number of awakenings	0.23	0.26	0.46	0.40	−0.62*	−0.38	−0.56*	0.18	0.24
Respiratory AI	−0.07	0.09	0.23	0.08	−0.36	−0.25	−0.30	0.33	0.28
Spontaneous AI	−0.11	−0.02	−0.30	−0.20	−0.27	−0.08	−0.21	−0.07	0.00
Total AI	−0.07	0.05	0.09	0.01	−0.18	−0.08	−0.19	0.06	−0.07
AHI	−0.18	−0.5	0.17	−0.06	−0.01	0.05	0.04	0.30	0.24
Supine AHI	−0.18	−0.05	0.17	−0.06	0.13	0.09	0.09	0.09	0.07
Central AHI	−0.22	0.14	−0.09	−0.13	0.04	0.05	0.04	0.27	0.27
RERA index	−0.14	−0.06	0.03	−0.10	−0.04	0.08	−0.04	0.03	−0.14
ODI	0.11	−0.01	0.310	0.183	−0.14	−0.19	−0.12	0.40	0.31
PLMI	−0.20	−0.07	0.12	−0.11	−0.03	−000	−0.05	−0.09	−0.15
PLMAI	−0.11	−0.01	0.12	−0.03	−0.10	−0.04	−0.11	−0.14	−0.21

AHI, apnea-hypopnoea index; AI, arousal index; HADS, Hospital Anxiety and Depression Scale; LEAS, Level of Emotional Awareness Scale; ME/CFS, myalgic encephalomyelitis/chronic fatigue syndrome; N1, N2, N3, respectively sleep stage 1, 2, 3; ODI, oxygen desaturation index; PLMAI, periodic limb movements arousal index; PLMI, periodic limb movements index; PSG, polysomnography; REM, rapid eye movements; RERA, respiratory effort related arousals; Toronto Alexithymia Scale (TAS-20) (DIF, difficulty in identifying feelings; DDF, difficulty in describing feelings; EOT, externally orientated thinking).

Correlations with p-values <0.001 are marked in bold.

There were two weak negative correlations between sleep efficiency and HADS depression (r = −0.44, p = 0.04) and anxiety (r = −0.43, p = 0.04), respectively, **Table 4**.

## Discussion

The focus of this study was the correlation analysis between parameters of sleep from polysomnograhy and emotional regulation evaluated with validated questionnaires TAS-20, LEAS, and HADS, respectively. The main results were the significant negative correlations between LEAS-self and LEAS-total and number of awakenings in polysomnography. This indicate that the awakenings and sleep fragmentation might be an important sleep disturbance, affecting emotional awareness. The awakenings could to some extent be explained by the fact that several patients had milder forms of other sleep disorders, for example OSA, periodic limb movement, and or restless legs. Interestingly, another study has shown that the severity of the OSA does not directly correlate with the severity of mood symptoms. Using the becks depression inventory (BDI) and State-Trait Anxiety Inventory Lee and colleagues investigated anxiety and depression in association with OSA severity (26). Interestingly, anxiety and depressive symptoms were more prevalent in patients with mild OSA than those with severe OSA. However, there were no other significant correlations between TAS-20 or HADS and polysomnography parameters, but this is only a pilot study with a small study sample, therefore no conclusions can be drawn. In a previous study, alexithymia measured by TAS-20 was associated with shorter pre-REM sleep stages (N1 and N3) in normal healthy participants (27). At the same time an increase in REM episodes but not in REM duration was associated with alexithymia (27).

In the present study, the different correlation results between polysomnography measures and TAS-20 vs. LEAS, indicate that alexithymia and emotional awareness could have different associations with sleep, and probably are regulated in different ways, though overlapping each other. This is also in line with our previously published meta-analytic review on correlation patterns between TAS-20 and LEAS in different psychiatric, psychosomatic, and somatic disorders (17). It is known that arousals and awakenings contribute to daytime sleepiness (18). However, the research on the role of arousals and awakenings is not so easy to perform since most of the studies are carried out on sleep deprivation in healthy subjects or animals, which is not the same as pathological arousals due to long-term disease status (28). The experiments with sleep deprivation in healthy subjects have demonstrated negative effects on mainly facial expression (29). As suggested, sleep deprivation amplifies amygdala reactivity in response to negative emotional stimuli, triggers emotional sensitivity, increases central sympathetic tonus, and disrupts peripheral autonomic nervous system feedback to visceral body information (29). Sleep is crucial in normalizing central adrenergic signaling, which usually occurs during REM sleep (30). In other words, there is a connection between long-term sleep disturbances, impaired emotional regulation and psychosomatic symptoms through several regulatory mechanisms. This connection might be in part applicable to ME/CFS patients, since clinically there is a broad panorama of both mental and somatic symptoms as well as increased tonus in the sympathetic nervous system.

In general, emotional regulation is described by identification of an emotion to be regulated; selection of emotion regulation strategy and implementation of this strategy (31). Emotional awareness and alexithymia are, therefore, crucial for identification of an emotion as well as placing it in a conscious context before applying the regulatory strategies. Research on sleep disorders and emotional awareness is scarce, but one study has indeed reported that mindfulness promotes better emotional regulation and improves sleep (32). Evaluation of healthy controls is also important since both insomnia (33) and alexithymia (34) are found in the normal population.

### Limitations and Strengths of the Study

Limitations of this study are the absence of sleep recordings in healthy controls, and a small study sample with no previous power analysis. The correlation analysis with a strict limit for significant p values might result in false negative results, and also multiple correlations might create positive false results randomly.

There are also several strengths: namely, the use of gold standard polysomnography, validated questionnaires, well-defined and thoroughly investigated ME/CFS patients.

## Conclusion

Our pilot study could indicate a biological mechanism such as sleep fragmentation, affecting emotional awareness. Further studies on emotional regulation should include both healthy participants and patient populations, even those with other psychosomatic and neurological conditions known to have symptomatology of sleep disorders. Furthermore, there is a need of more objective evaluation of emotional regulation, since TAS-20 and LEAS carry a risk of learning moment when applied several times. Altogether, future research is important to explore our preliminary results, including treatment strategies on both sleep disorders and emotional regulation in order to understand the connection between sleep and emotions.

## Data Availability Statement

The datasets generated for this study are available on request to the corresponding author.

## Ethics Statement

The studies involving human participants were reviewed and approved by the regional ethical review board in Stockholm (Ref. no. 2014/300-31) and approved by Danderyd University Hospital (DS2014-0447). The patients/participants provided their written informed consent to participate in this study.

## Author Contributions

IB-L has put together and analyzed the results of sleep parameters and emotional regulation. She wrote the main draft of the manuscript and created the tables. DF performed the major job in analyzing the sleep protocols and reviewing the manuscript with tables.

## Conflict of Interest

The authors declare that the research was conducted in the absence of any commercial or financial relationships that could be construed as a potential conflict of interest.
